# Supplementary figures and images for: EZH2 Is Essential for Fate Determination in the Mammalian Isthmic Area
Source: Front Mol Neurosci. 2019 Apr 9;12:76. doi: 10.3389/fnmol.2019.00076 (PMC6465967; doi:10.3389/fnmol.2019.00076)

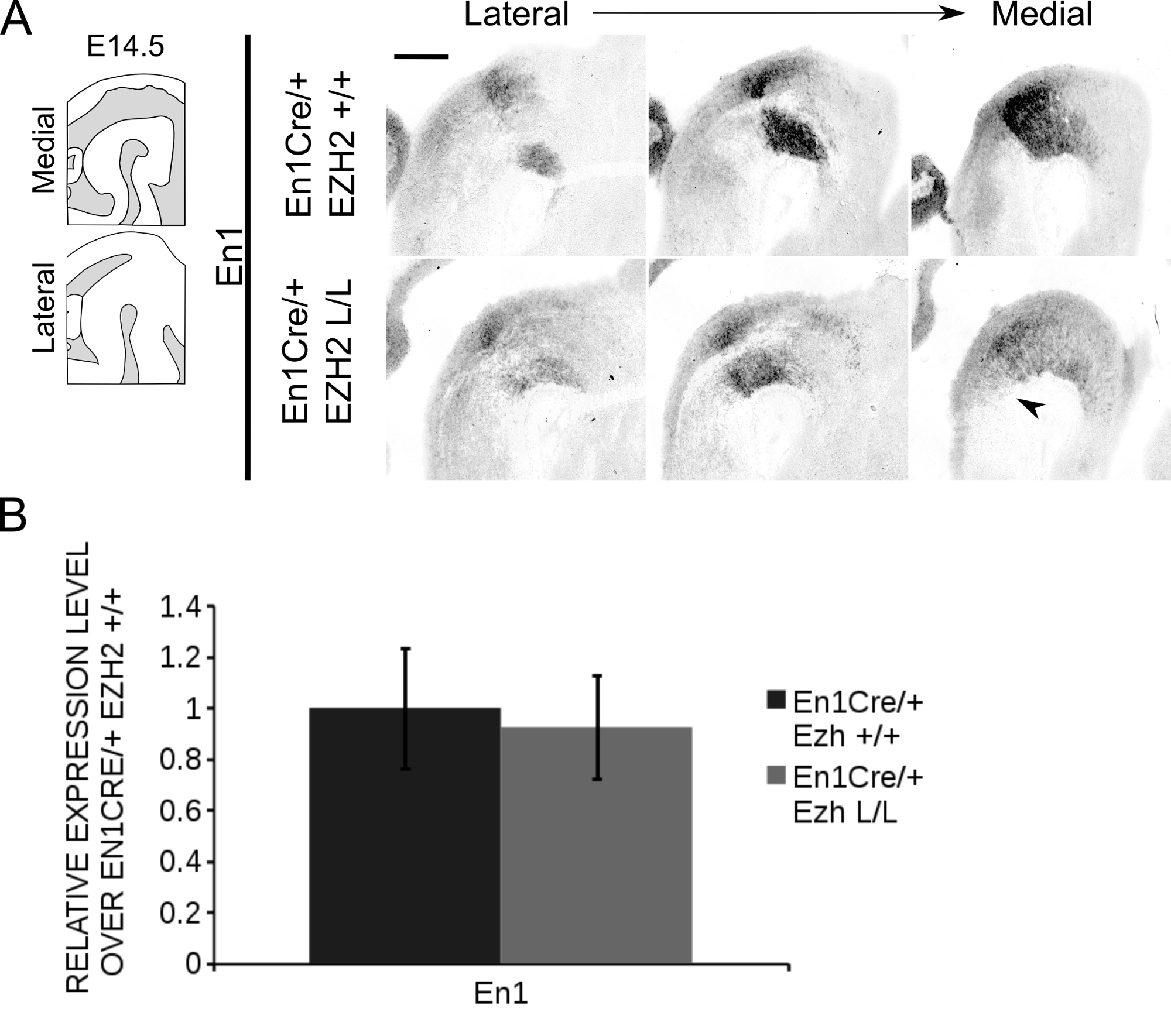

Supplement: FIGURE S1 — The loss of Ezh2 does not influence En1 expression. The expression of En1 was analyzed with in situ hybridization and qPCR at E14.5 (A, B). (A) En1 shows a similar expression pattern between the En1Cre/+; Ezh2 +/+ and the En1Cre/+; Ezh2 L/L animals. However ectopic expression of En1 might be present in the ventral region of R1 (arrowhead). (B) The levels of En1 are not significantly changed between wildtypes and Ezh2 cKO animals at E14.5. Scale bar = 300 μM. [file Image_1.TIFF]

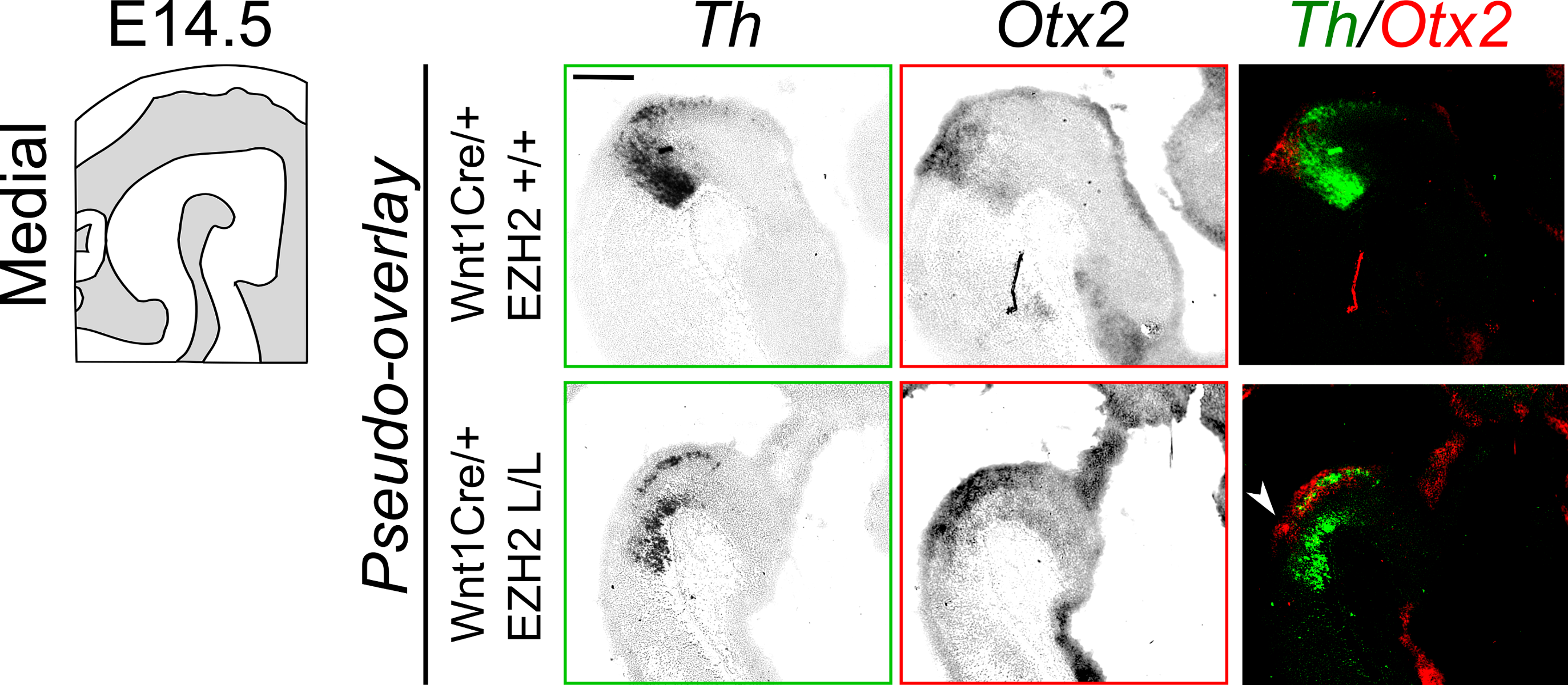

Supplement: FIGURE S2 — Otx2 is found caudal to the midbrain in Wnt1Cre/+; Ezh2 L/L embryos. Otx2 expression was assessed by means of in situ hybridization using Th expression as a reference for the location. A pseudo-overlay of adjacent slides demonstrates the presence of Otx2 expression dorsal of the ectopic Th expression in medial sections of Wnt1Cre driven Ezh2 cKOs (arrowhead). Scale bar = 300 μM. [file Image_2.TIFF]

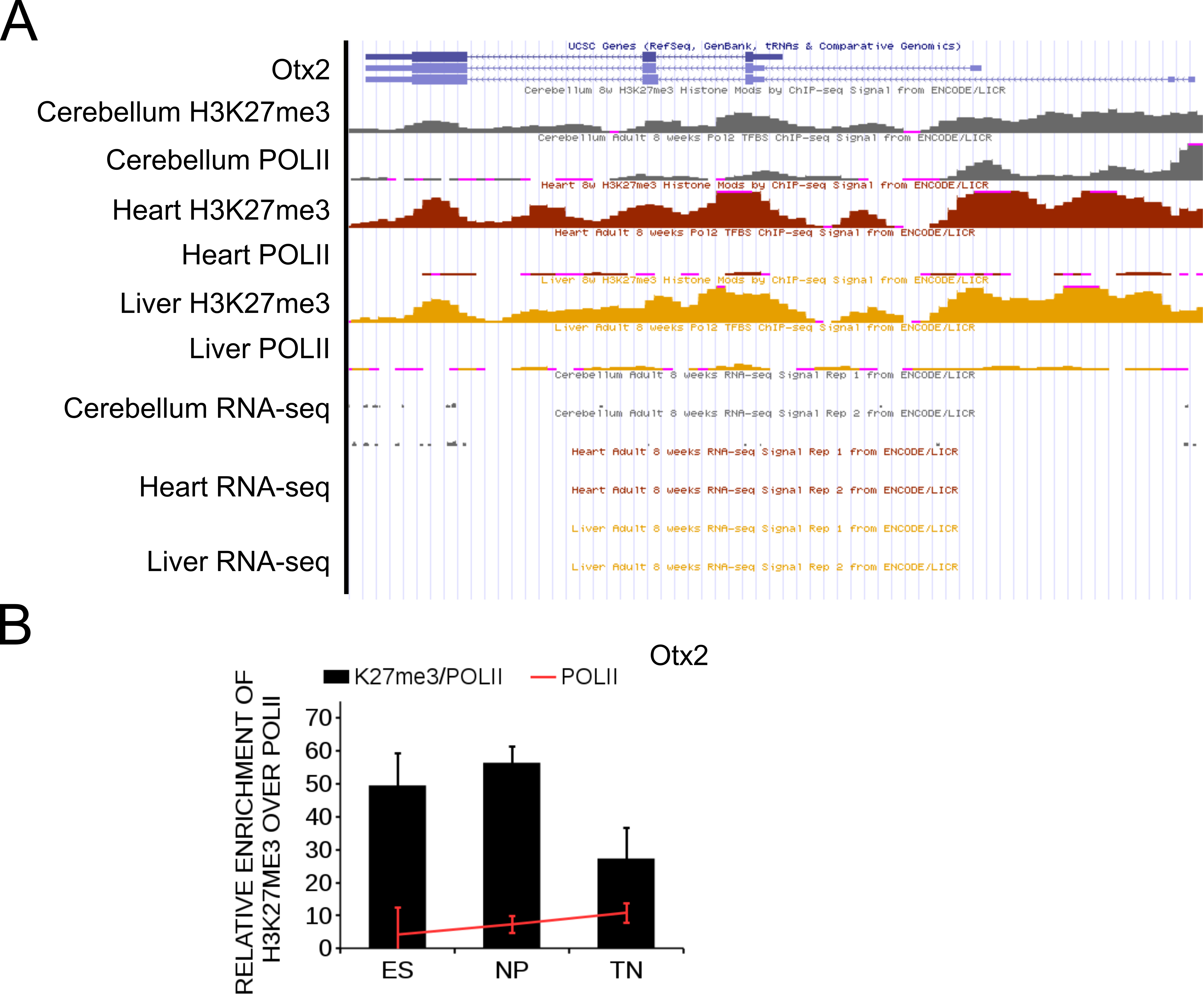

Supplement: FIGURE S3 — H3K27me3 is present on the promoter of Otx2 in tissue in which Otx2 is not expressed. (A) Visualization of ChIP-sequencing peaks of H3K27me3 and RNA-polymerase II (POLII) at the Otx2 promoter obtained from https://genome.ucsc.edu/index.html using the mouse NCBI37/mm9 assembly. In tissue where almost no RNA-sequencing reads are detected for Otx2, a high peak for H3K27me3 is observed, while the peak for POLII is low. (B) Analysis of ChIP-sequencing data from Mohn et al. (2008). The Otx2 promoter shows a high enrichment for H3K27me3 in Embryonic stem cells (ES), Neuronal progenitors (NP) and Terminal pyramidal glutamatergic neurons (TN), while enrichment for POLII is low, indicating that Otx2 is not activated in these cells. [file Image_3.TIFF]
